# Supplementary material for: Polarization of the Drosophila oocyte by active cortical exchange
Source: ArXiv. 2026 Oct 7:arXiv:2610.10916v1. Preprint. [Version 1] (PMC13692671)
Supplement: Supplementary file 1 [file NIHPP2610.10916V1-supplement-1.pdf]

Supporting Information for: Polarization of the *Drosophila* oocyte by active cortical exchange

Mohammadhossein Firouznia[a], Wen Lu[b], Margot Lakonishok[b], Reza Farhadifar[a], Vladimir I. Gelfand[b], and Michael J. Shelley[a,c]

[a]Center for Computational Biology, Flatiron Institute, New York, NY 10010
[b]Department of Cell and Developmental Biology, Feinberg School of Medicine, Northwestern University, Chicago, IL 60611
[c]Courant Institute, New York University, New York, NY 10012

1 Anterior-to-posterior distance from stage 7 to stage 9

We quantified the changes in oocyte size by fixing $n = 18$ oocytes at stages 7–9 and staining them with DAPI. We measured the oocyte length as the distance from the anterior side (shared with the nurse cells) to the posterior pole in the mid-plane of the oocyte.

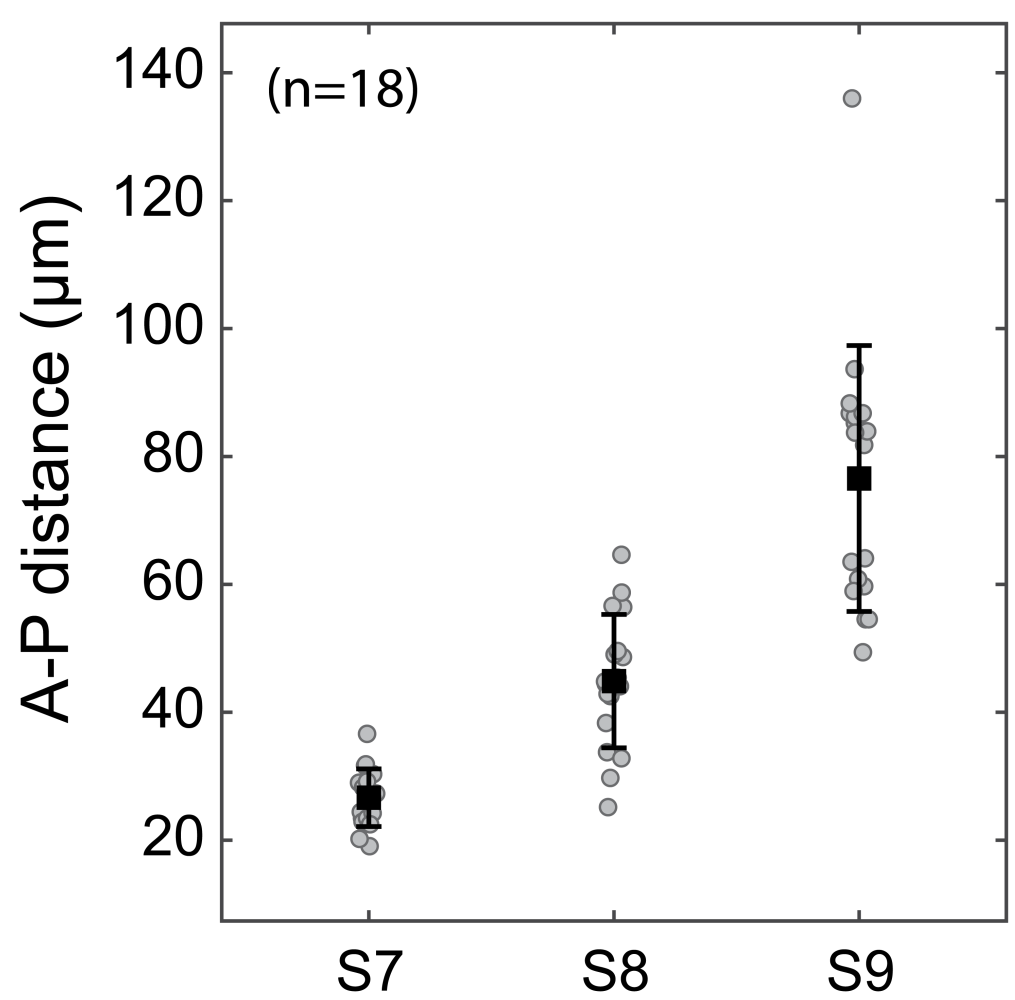

Figure S1: Length of A-P axis of oocytes at stage 7 (27 ± 5 μm, $n = 18$), stage 8 (45 ± 10 μm, $n = 18$), and stage 9 (77 ± 21 μm, $n = 18$). Values are mean $\pm$ one standard deviation.

2 Cortical Patronin profiles from stage 7 to stage 9

We measured the normalized cortical intensity profile of Patronin-GFP, $I_p(s)$, in stage 7 to 9 oocytes, where the arclength $-1 \leq s \leq 1$ is measured from the posterior pole ($s = 0$).

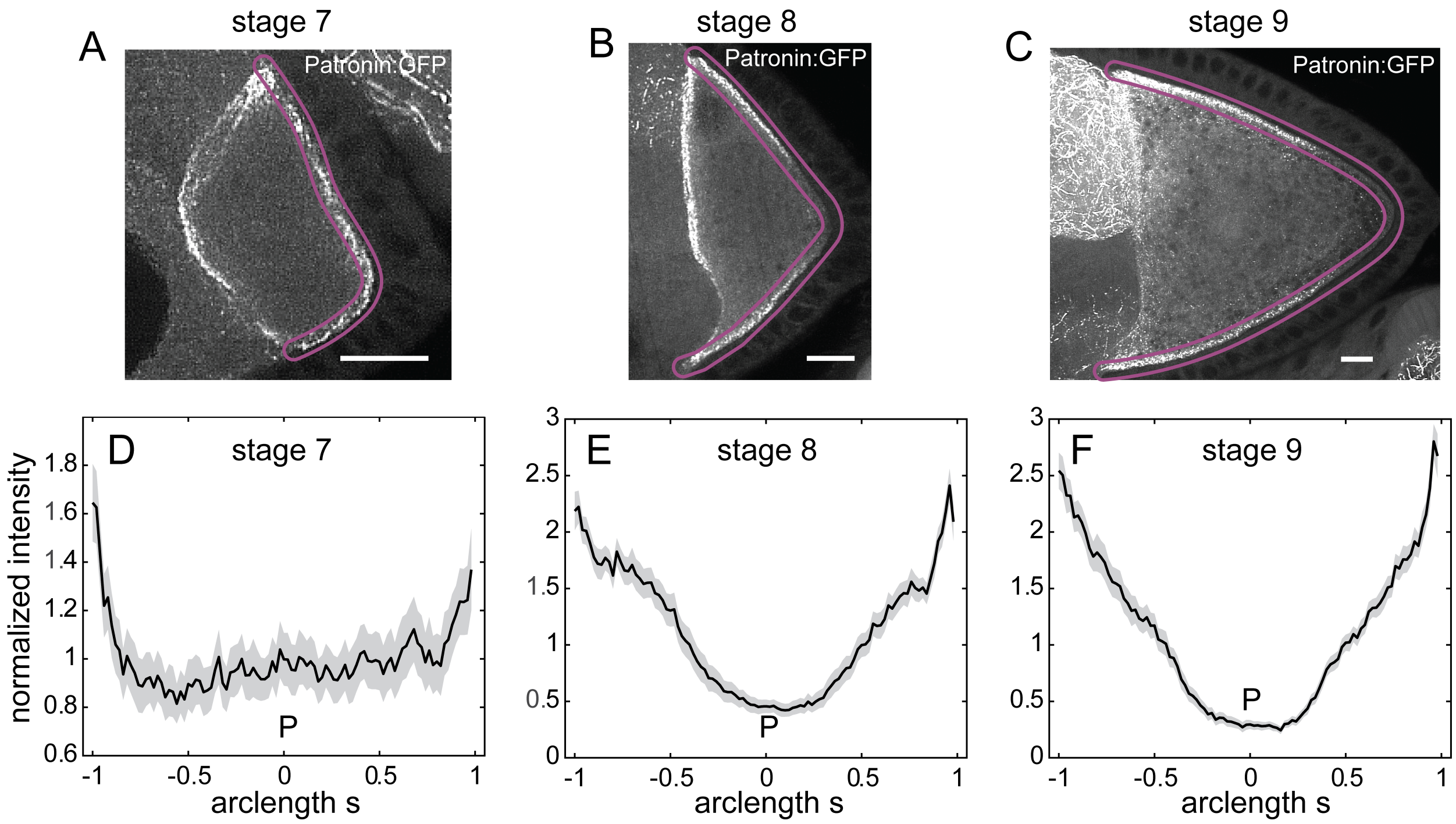

Figure S2: (A–C) Representative images of Patronin-GFP in the mid-plane of stage 7 (A), stage 8 (B), and stage 9 (C) oocytes; the cortical region is outlined in purple. Scale bar, 10 μm. (D–F) Surface-averaged Patronin intensity profile, I_p, as a function of the normalized cortical arclength s measured from the posterior pole, at stage 7 (D; $n = 35$), stage 8 (E; $n = 37$), and stage 9 (F; $n = 35$) (black line, mean; shaded region, standard error of the mean). The profile is normalized so that its average over the cortical surface is unity, $\int_{\partial V} I_p \, \mathrm{d}A \, / \int_{\partial V} \mathrm{d}A = 1$. The posterior pole (P) at $s = 0$ is indicated.

3 Model for Transport and Cortical Exchange of *oskar* mRNA

As discussed in the main text, we propose a continuum model for the cytoplasmic transport and cortical exchange of *oskar* during stages 7–9 of *Drosophila* oogenesis based on local thermodynamic equilibrium. The model describes two coupled processes: (i) phase separation of *oskar* and (ii) its reversible association with the cortex through binding and unbinding. We consider a binary mixture of *oskar* with concentration c and molecular volume ν in a solvent with an effective molecular volume ν_0 that represents the rest of the cytoplasmic fluid. The local composition of the mixture is therefore characterized by volume fraction $\phi = c\,\nu$, with $0 \leq \phi \leq 1$. The phase separation of *oskar* in the cytoplasm can be described by an effective free energy which is a function of local volume fraction ϕ and temperature T:

$$\mathcal{F}(\phi, T) = \int_V \mathrm{d}v \left[f(\phi, T) + \frac{\kappa}{2\,\nu_0} |\nabla\phi|^2 \right]. \tag{1}$$

Here, κ characterizes the change in free energy due to concentration inhomogeneities, and $f(\phi, T)$ is a binary Flory–Huggins free energy per unit volume given by [1–5]:

$$f(\phi, T) = k_B\,T \left[\frac{\phi}{\nu} \ln\phi + \frac{(1-\phi)}{\nu_0} \ln(1-\phi) + \frac{\chi}{\nu_0}\,\phi(1-\phi) \right]. \tag{2}$$

The effective interactions among *oskar* molecules and between *oskar* and the solvent are characterized by $\chi(T)$, with $\chi > 0$ promoting demixing. In our model, we consider a fixed spherical domain with radius R. The diffusive flux of *oskar* is given by $\boldsymbol{J} = -M\boldsymbol{\nabla}\mu$, where $\mu = \nu\,\delta\mathcal{F}/\delta\phi$ is the exchange chemical potential, and $M = m\,\phi(1-\phi)$ is the mobility describing the molecular exchange of *oskar* with the solvent with a probability proportional to *oskar* and solvent volume fractions. The evolution of ϕ obeys the conservation of fluxes of *oskar*:

$$\partial_t\phi + \boldsymbol{\nabla}\,.\,\boldsymbol{J} = 0, \qquad \boldsymbol{x} \in V. \tag{3}$$

Defining $D = m\,k_B T$, $\gamma = \kappa\,m\,\lambda$, $\lambda = \nu/\nu_0$, the transport equation becomes:

$$\partial_t\phi = D\,\Delta\big[\phi + g(\phi)\big] - \gamma\,\boldsymbol{\nabla}\cdot\big[\phi(1-\phi)\,\boldsymbol{\nabla}(\Delta\phi)\big], \qquad \boldsymbol{x} \in V, \tag{4}$$

$$g(\phi) = \Big[(\lambda - 2\,\lambda\,\chi - 1)\frac{\phi^2}{2} + 2\lambda\,\chi\,\frac{\phi^3}{3}\Big]. \tag{5}$$

The parameters λ and χ are inferred from experimental measurements, as described in Secs. 5 and 6. The boundary conditions are:

$$\boldsymbol{n}\,.\,\boldsymbol{J} = \ell_c\big[\beta\,\phi - \alpha\,\phi_c\big], \qquad \boldsymbol{x} \in \partial V, \tag{6}$$

$$\partial_t\phi_c = \beta\,\phi - \alpha\,\phi_c, \qquad \boldsymbol{x} \in \partial V, \tag{7}$$

$$\boldsymbol{n}\,.\,\boldsymbol{\nabla}\phi = 0, \qquad \boldsymbol{x} \in \partial V, \tag{8}$$

where ϕ_c is the volume fraction of surface-bound *oskar* in a cortical layer of thickness ℓ_c. β is the uniform binding rate due to myosin-V activity, and α is the position-dependent unbinding rate due to kinesin-1 activity. Eq. 6 balances the flux of *oskar* at the cortex with the net exchange due to cortical binding and unbinding. According to Eqs. 6 and 7, the total amount of *oskar* is conserved in the system:

$$\frac{\mathrm{d}}{\mathrm{d}t}\Big[\ell_c \int_{\partial V} \phi_c(t)\,\mathrm{d}A + \int_V \phi(x,t)\,\mathrm{d}V\Big] = 0. \tag{9}$$

The unbinding rate varies along the cortex following the Patronin density distribution that reflects microtubule density gradients:

$$\alpha(\boldsymbol{x}) = \alpha_{\text{SF}}\, I_{\text{p}}(\boldsymbol{x}), \qquad \boldsymbol{x} \in \partial V, \tag{10}$$

where I_{p} is the normalized density distribution of Patronin, given in Fig. 3 of the main text. Eq. 7 tracks the net accumulation of cortex-bound *oskar* while Eq. 8 imposes a neutral wetting condition, ensuring no additional affinity between the *oskar*-rich phase and the cortex. Together, Eqs. 6 and 8 are equivalent to:

$$\boldsymbol{n} \cdot \boldsymbol{\nabla}(\Delta\phi) = \frac{\ell_c}{\gamma}\Big[\frac{\beta\,\phi - \alpha\,\phi_c}{\phi(1-\phi)}\Big], \qquad \boldsymbol{x} \in \partial V. \tag{11}$$

Nondimensionalizing the governing equations using R as the length scale and $t_d = R^2/D$ as the time scale yields, in addition to χ and λ, the dimensionless groups

$$A = \alpha_{\text{SF}}\, t_d, \qquad B = \beta\, t_d, \qquad \epsilon = \frac{\ell_c}{R}, \qquad \Gamma = \frac{\gamma\, t_d}{R^4}. \tag{12}$$

The nondimensional governing equations are:

$$\partial_t\phi = \psi + \Delta g(\phi) - \Gamma\,\boldsymbol{\nabla}\cdot\big[\phi(1-\phi)\,\boldsymbol{\nabla}\psi\big], \qquad \boldsymbol{x} \in V, \tag{13}$$

$$\psi = \Delta\phi, \tag{14}$$

$$\boldsymbol{n} \cdot \boldsymbol{\nabla}\psi = \frac{\epsilon}{\Gamma}\Big[\frac{B\,\phi - A\,\phi_c}{\phi(1-\phi)}\Big], \qquad \boldsymbol{x} \in \partial V, \tag{15}$$

$$\partial_t\phi_c = B\,\phi - A\,\phi_c. \qquad \boldsymbol{x} \in \partial V, \tag{16}$$

$$\boldsymbol{n} \cdot \boldsymbol{\nabla}\phi = 0, \qquad \boldsymbol{x} \in \partial V. \tag{17}$$

For the analysis of photoconversion experiments in Sec. 4, we take the limit $\chi, \Gamma \to 0$ and $\lambda \to 1$, in which Eqs. 13–17 reduce to simple diffusion in the bulk with the continuity of flux at the boundary:

$$\partial_t\phi = \Delta\phi, \qquad \boldsymbol{x} \in V, \tag{18}$$

$$\boldsymbol{n} \cdot \boldsymbol{\nabla}\phi = \epsilon\,\big[A\,\phi_c - B\,\phi\big], \qquad \boldsymbol{x} \in \partial V, \tag{19}$$

$$\partial_t\phi_c = B\,\phi - A\,\phi_c. \qquad \boldsymbol{x} \in \partial V. \tag{20}$$

We solve Eqs. 13–17 numerically using the `Dedalus` spectral PDE solver in a spherical geometry of radius R [6]. The linear terms are treated implicitly, while the nonlinear terms are treated explicitly. The initial condition is a uniform bulk volume fraction $\phi(\boldsymbol{x}, 0) = \phi_0$ with zero cortex-bound volume fraction $\phi_c(0) = 0$. The total amount of *oskar* is thus set by the initial volume fraction ϕ_0, a key parameter controlling the phase-separation behavior. The ratio of unbinding to binding rates, or the exchange equilibrium constant, $r_{\text{ex}} = A/B = \alpha_{\text{SF}}/\beta$, determines the ratio of bulk to cortex-bound volume fractions at equilibrium, whereas the individual values of A and B affect only the time to reach equilibrium. The cortical layer thickness is measured to be $\ell_c \approx 10$ μm at the posterior pole, which yields $\epsilon \approx 0.25$ (with $R_{\text{oocyte}} = R + \ell_c \approx 50$ μm at stage 9). The parameter Γ controls the stability of the system against phase separation (increasing Γ opposes phase separation) and sets the length scale of gradients in ϕ. We set $\Gamma = B = 1$ and vary A and ϕ_0 to construct the phase diagram in Fig. 4 of the main text.

4 Photoconversion of Staufen

In photoconversion experiments, an area of approximately 550 μm^2 is exposed to ultraviolet light. Staufen-MoxMaple3 switches irreversibly from green to red fluorescence upon exposure, allowing a distinct population of Staufen to be labeled and tracked simultaneously over time across three distinct cortical regions: the anterior, mid-lateral, and posterior cortex. Red fluorescence intensity is averaged over the box. We observe that variations in fluorescence intensity are predominantly along the direction normal to the cortex. Inspired by this observation, we use the model described in Sec. 6 in one dimension. The time span of photoconversion is 10 min, which is much shorter than that of oogenesis from stage 7 to 9 (~ 12 hours), where *oskar* RNPs aggregate and phase separate in the bulk. We therefore neglect the interactions among *oskar* RNPs, and between *oskar* RNPs and the solvent, that drive demixing in the bulk ($\chi = 0$). In the absence of strong inhomogeneities in the bulk, we also neglect the corresponding gradient free energy ($\kappa = 0$). The model then reduces to a diffusion equation on the half-line, subject to cortical binding and unbinding at the boundary:

$$\partial_t \phi = D\, \partial_x^2 \phi, \qquad x \geq 0, \tag{21}$$

$$D\, \partial_x \phi = \ell_c \big[\beta\, \phi(0,t) - \alpha\, \phi_c(t)\big], \qquad x = 0, \tag{22}$$

$$\partial_t \phi_c = \beta\, \phi(0,t) - \alpha\, \phi_c(t), \qquad x = 0, \tag{23}$$

where ϕ is the volume fraction of *oskar* in the bulk and ϕ_c is the volume fraction of cortically bound *oskar* within a layer of thickness ℓ_c. The constants β and α are the binding and unbinding rates (units of s^{-1}). The initial conditions are $\phi(x,0) = \phi_0\,[H(x+\ell_b) - H(x-\ell_b)]$ and $\phi_c(0)$, where $H(x)$ is the Heaviside step function; on the half-line the initial bulk profile is a box of height ϕ_0 on $0 \leq x \leq \ell_b$. The exposed area 550 μm^2 corresponds to a square of side $\ell = 23$ μm, with $\ell = \ell_b + \ell_c$.

We introduce the Laplace transform in time, $\hat{\phi}(x,s) = \int_0^\infty e^{-st}\, \phi(x,t)\, dt$, and define $q = \sqrt{s\,\ell_b^2/D}$. The diffusion equation becomes an ordinary differential equation in x in Laplace space with the initial condition as a source term, and the cortical equation transforms algebraically:

$$D\, \hat{\phi}_{xx} - s\, \hat{\phi} = -\phi(x,0), \tag{24}$$

$$\hat{\phi}_c(s) = \frac{\beta\, \hat{\phi}(0,s) + \phi_c(0)}{s+\alpha}. \tag{25}$$

The bulk volume fraction is a piecewise function,

$$\hat{\phi}(x,s) = \begin{cases} \dfrac{\phi_0}{s} + C_1\, e^{qx/\ell_b} + C_2\, e^{-qx/\ell_b}, & 0 \leq x/\ell_b \leq 1, \\ C_3\, e^{-qx/\ell_b}, & x/\ell_b \geq 1, \end{cases} \tag{26}$$

Substituting Eq. (25) and Eq. (26) into the boundary condition Eq. (22), and enforcing the continuity of $\hat{\phi}$ and $\partial_x \hat{\phi}$ at $x = \ell_b$, we determine the coefficients:

$$C_1 = -\frac{\phi_0}{2s}\, e^{-q}, \qquad C_2 = \hat{\phi}(0,s) - \frac{\phi_0}{s} + \frac{\phi_0}{2s}\, e^{-q}, \qquad C_3 = \hat{\phi}(0,s) + \frac{\phi_0}{s}(\cosh q - 1), \tag{27}$$

where the boundary value is

$$\hat{\phi}(0,s) = \frac{\phi_0\, \sqrt{D/s}\,(1 - e^{-q}) + \ell_c\, \dfrac{\alpha\, \phi_c(0)}{s+\alpha}}{\sqrt{Ds} + \ell_c\, \dfrac{\beta s}{s+\alpha}}. \tag{28}$$

Setting $\beta = 0$ and $\phi_c(0) = 0$ reduces the boundary value to $\hat{\phi}(0, s) = \phi_0\,(1 - e^{-q})/s$, which inverts to $\phi(0, t) = \phi_0\,\mathrm{erf}(\ell_b/(2\sqrt{Dt}))$, the boundary value under a no-flux boundary condition. In this limit, $C_1 = C_2$ and the solution reduces to the symmetric spreading of the box-shaped initial condition.

We determine the box-averaged volume fraction of *oskar* in a window of size ℓ over time. We compare its normalized value with the normalized fluorescent intensity obtained in photoconversion experiments:

$$\bar{\phi}(t) = \frac{1}{\ell}\left[\,\ell_c\,\phi_c(t) + \int_0^{\ell_b} \phi(x, t)\,\mathrm{d}x\right], \tag{29}$$

$$\bar{\phi}(0) = \frac{1}{\ell}\left[\,\ell_c\,\phi_c(0) + \phi_0\,\ell_b\,\right], \tag{30}$$

$$I_{\mathrm{obs}}(t) = \frac{\bar{\phi}(t)}{\bar{\phi}(0)}. \tag{31}$$

The bulk contribution to Eq. (29) is

$$\int_0^{\ell_b} \hat{\phi}(x, s)\,\mathrm{d}x = \frac{\phi_0\,\ell_b}{s} + \frac{\ell_b}{q}\Big[\,C_1\,(e^{q} - 1) + C_2(1 - e^{-q})\,\Big], \tag{32}$$

in Laplace space. The boundary value $\hat{\phi}(0, s)$ in Eq. (28) does not have an elementary inverse transform, due to the $\sqrt{s}$ term that appears with the rational reaction factor $\beta s/(s + \alpha)$ in the denominator. Therefore, $\phi(x, t)$, $\bar{\phi}(t)$, and $I_{\mathrm{obs}}(t)$ are recovered by numerical Laplace inversion [7]. The rates α, β, D/ℓ^2 and the thickness ratio ℓ_b/ℓ are inferred by fitting $I_{\mathrm{obs}}(t)$ to the measured intensity decay. Fig. S3 shows the photoconversion intensity decay averaged over 18 oocytes, with the least-squares fit of the model overlaid as solid lines at each cortical position. Fig. S4 shows the distributions of fitted parameters across the same 18 oocytes, and Table S1 lists the mean, standard deviation, and standard error of the mean for each parameter. Based on experimental measurements at $t = 0$ s, the cortical volume fraction exceeds the bulk volume fraction at the posterior pole. We therefore set $\phi_c(0)/\phi_0 = 2$ at the posterior pole, and $\phi_c(0)/\phi_0 = 1$ at the anterior pole and mid-lateral point.

Table S1: Fitted parameters from photoconversion experiments (mean ± SD across $n = 14$ experiments). Parameters D/w^2 and β are constant across cortical positions, while α and ℓ_b/ℓ are position-dependent.

Parameter	Description	Mean	SD
D/w^2 (min^{-1})	Diffusion rate	1.01	0.30
β (min^{-1})	Binding rate	2.61	1.09
α_a (min^{-1})	Unbinding rate, anterior	1.15	0.57
α_m (min^{-1})	Unbinding rate, mid-lateral	0.80	0.42
α_p (min^{-1})	Unbinding rate, posterior	0.24	0.17
$(\ell_b/\ell)_a$	Bulk fraction, anterior	0.79	0.073
$(\ell_b/\ell)_m$	Bulk fraction, mid-lateral	0.70	0.084
$(\ell_b/\ell)_p$	Bulk fraction, posterior	0.84	0.048

The optical section thickness in spinning-disk confocal microscopy is much smaller than the lateral field of view, $w \approx 1$ μm $\ll \ell \approx 23$ μm. Consequently, *oskar* RNPs diffuse out of the imaging plane faster than they traverse the field of view laterally. Therefore, the observed fluorescence decay

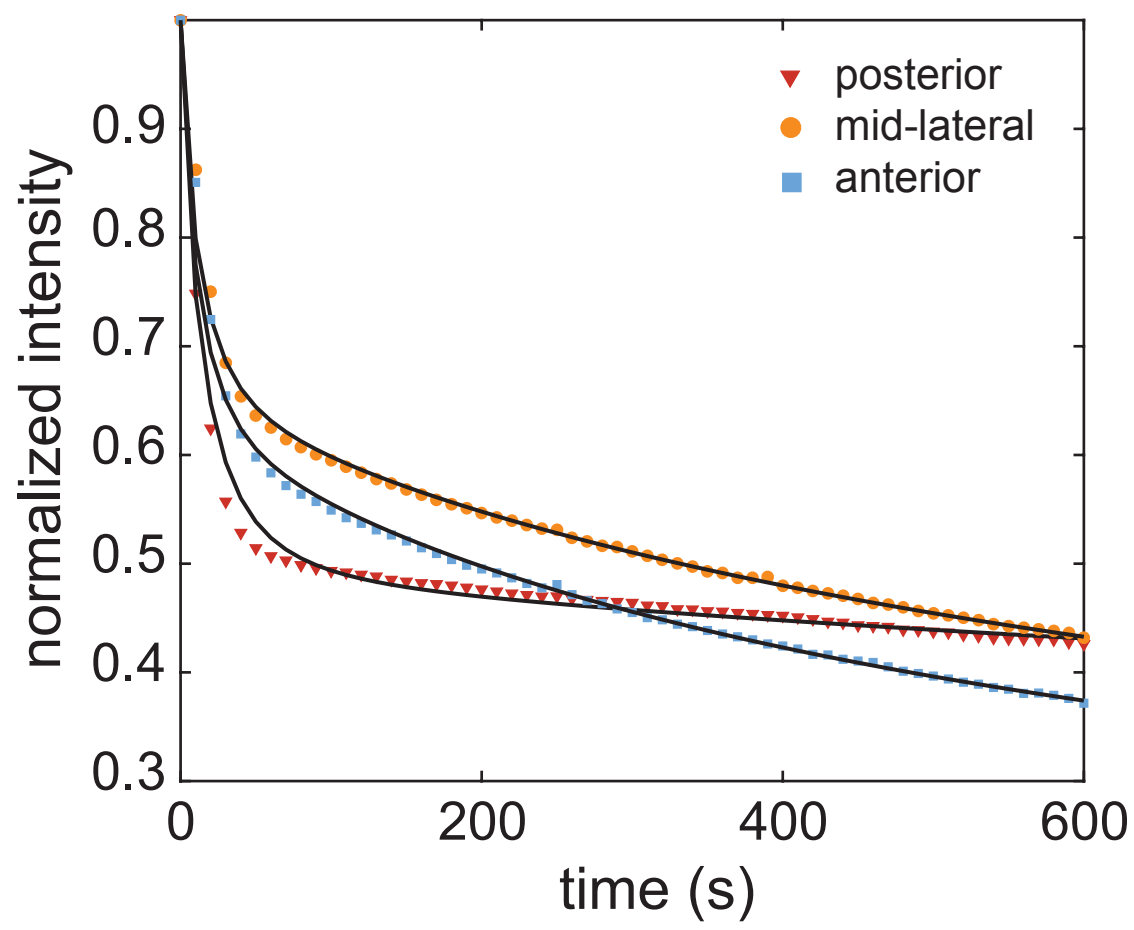

Figure S3: Fluorescence intensity at three cortical positions in photoconversion experiments shown by markers ($n = 18$). Solid lines are the least-squares fits of the model with $(D/w^2,\ \beta) = (0.0144,\ 0.0406)$ (s^{-1}). The fitted unbinding rates are $(\alpha_p, \alpha_m, \alpha_a) = (0.00442, 0.0144, 0.0203)$ (s^{-1}) and the bulk-thickness ratios are $((\ell_b/\ell)_p,\ (\ell_b/\ell)_m,\ (\ell_b/\ell)_a) = (0.847,\ 0.703,\ 0.793)$ at the posterior, mid-lateral, and anterior regions, respectively.

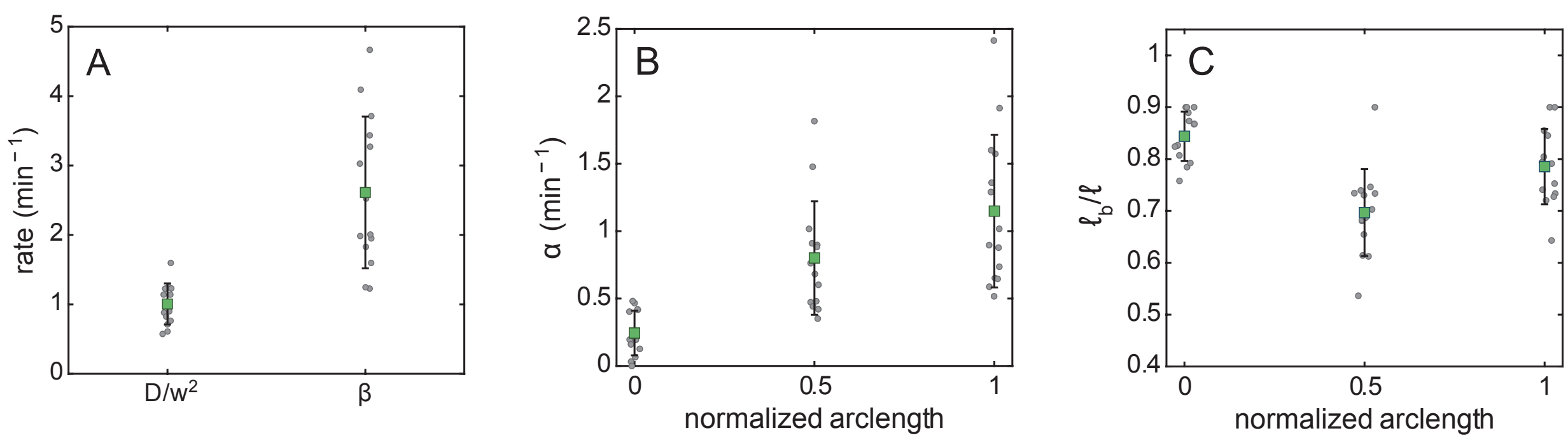

Figure S4: Fit parameters of the model for photoconversion experiments ($n = 14$): (A) Diffusion rate D/w^2 and binding rate β are constant across cortical positions, while (B) the unbinding rate α and (C) the bulk thickness ratio ℓ_b/ℓ are position-dependent.

is dominated by out-of-plane diffusion. Taking the cytoplasm to be ~ 1000 times more viscous than water, we estimate the effective particle radius from the Stokes-Einstein relation together with the fitted diffusion rate in Table S1:

$$\frac{k_B T}{3\pi\, \mu\, a_{\text{eff}}\, (w/2)^2} = 1\ \text{min}^{-1}, \tag{33}$$

$$a_{\text{eff}} \approx 100\ \text{nm}, \tag{34}$$

which is comparable to the average *oskar* RNP radius of 200 nm reported in [8].

5 Measuring the total *oskar* mRNA in the oocyte

To estimate the number of *oskar* mRNA granules at the posterior cap, *oskar* was visualized by single-molecule inexpensive fluorescence in situ hybridization (smiFISH), and images were acquired as non-saturating z-stacks. For each oocyte, a single optical section containing clearly resolved cytoplasmic *oskar* granules was selected from the z-stack to estimate the fluorescence intensity of individual particles. Single *oskar* granules were detected using the Find Maxima function in Fiji/ImageJ, with the noise threshold adjusted for each image to match visually identifiable single granules. Because the xy pixel size was approximately 200 nm, which is close to the expected size of *oskar* granules, each local maximum detected by the Find Maxima function was treated as one *oskar* granule. The fluorescence intensity of the pixels detected by the Find Maxima function was measured, and the average intensity of dim single particles was estimated by excluding the five lowest values and averaging the next 20 lowest mean-intensity values. To quantify posterior-cap *oskar*, the full *oskar* z-stack was sum-projected. Background fluorescence was measured from three regions outside the egg chamber and averaged. The background-corrected total fluorescence intensity of the entire oocyte was calculated by subtracting the background signal from the oocyte signal. The estimated number of *oskar* granules in the entire oocyte was then calculated by dividing this background-corrected oocyte total intensity by the average intensity of dim single cytoplasmic *oskar* granules (Fig.S5). The estimated *oskar* mRNA granule abundance scales with oocyte size.

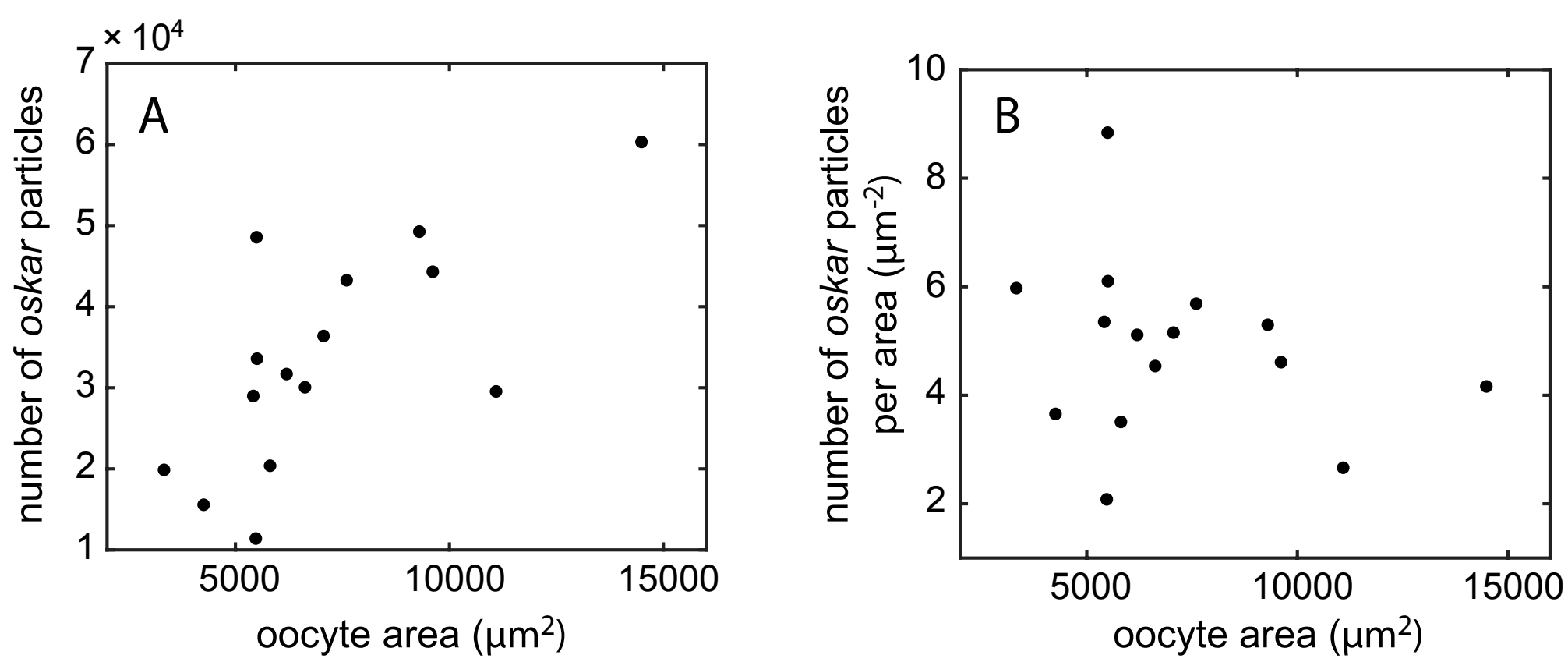

Figure S5: (A) Estimated total number of *oskar* mRNA particles plotted against oocyte area. Particle number was calculated by dividing the background-corrected total fluorescence intensity of each oocyte by the mean fluorescence intensity of a single cytoplasmic *oskar* particle. (B) Estimated *oskar* particle density, calculated as particle number per unit oocyte area, plotted against oocyte area. Each dot represents one oocyte.

6 Fitting of thermodynamic model to the experiments

$$f(\phi, T) = k_B T \left[\frac{\phi}{\nu} \ln \phi + \frac{(1-\phi)}{\nu_0} \ln(1-\phi) + \frac{\chi}{\nu_0} \phi(1-\phi) \right]. \tag{35}$$

$$\mu_{\text{in}} = \mu_{\text{out}}, \tag{36}$$

$$f_{\text{in}} - \mu_{\text{in}} \frac{\phi_{\text{in}}}{\nu} = f_{\text{out}} - \mu_{\text{out}} \frac{\phi_{\text{out}}}{\nu} + \frac{2\gamma}{R}, \tag{37}$$

Table S2 lists the parameters used to determine the thermodynamic properties of *oskar*–Staufen particles. The mean number of particles N and mean oocyte cross-sectional area A at stage 9 are measured in Fig. S5, and the mean particle radius a is taken from [8]. Together with the sample thickness h, these yield estimates of the total particle concentration c_0, total volume fraction ϕ_0, and the ratio ν/ν_0, where $\nu_0 = 18.015 \times 10^{-3}$ M^{-1} is the molar volume of water.

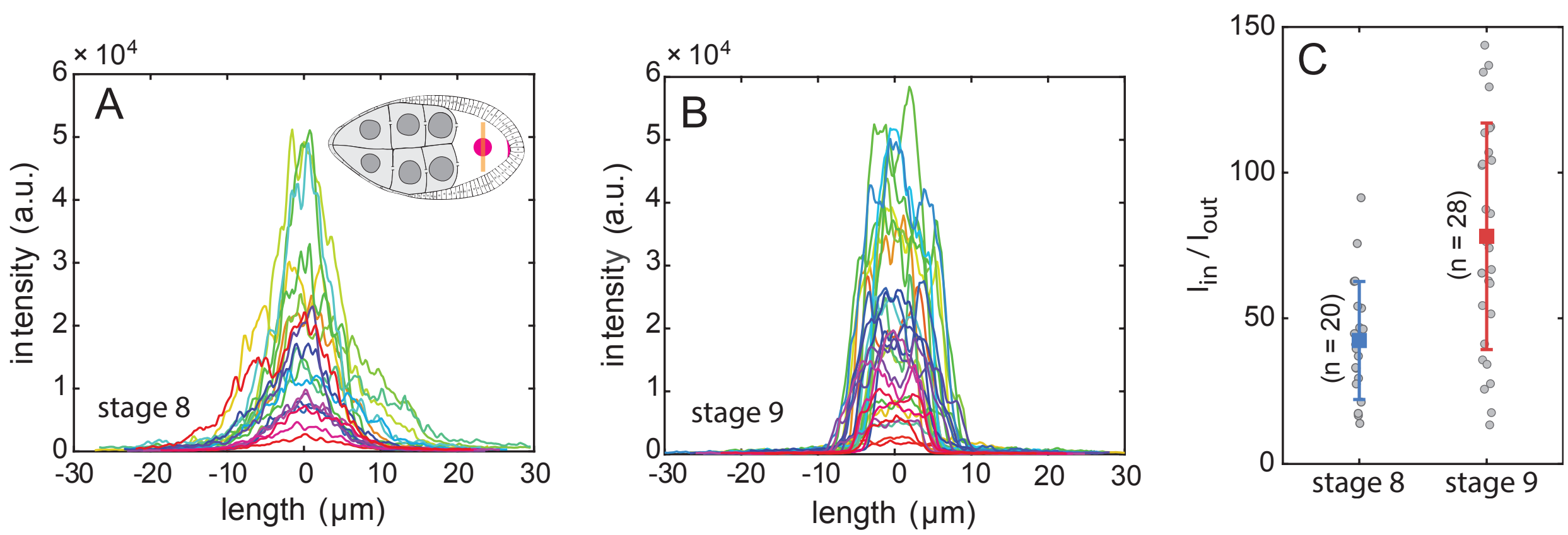

Figure S6: Intensity profile along a line (thickness 7–10 pixels, with 5.2 pixels = 1 μm) perpendicular to the anterior–posterior axis, passing through the phase-separated droplet as shown in the inset, under Patronin overexpression at (A) stage 8 and (B) stage 9. (C) Intensity ratio $I_{\text{in}}/I_{\text{out}}$ at each stage, with mean values and standard deviations 42 ± 20 at stage 8 ($n = 20$), and 78 ± 39 at stage 9 ($n = 28$). Error bars represent one standard deviation, and n denotes the number of oocytes analyzed. The p value between the two groups is $p = 0.0005$.

In Fig. S6A,B we measured the fluorescence intensity profile along a line through a phase-separated droplet at stages 8 and 9. For each oocyte, we first identified the z-slice of maximum *oskar* intensity and drew a line of 10 px width through the droplet, perpendicular to the anterior–posterior axis. We extracted the intensity profile along this line at the three adjacent z-slices. The intensity inside the droplet was estimated by averaging over pixels above 80% of the maximum. Taking the center of this high-intensity region as a reference, the outside intensity was estimated by averaging over positions more than 20 μm away, well beyond the droplet radius of $\lesssim$ 10 μm at stage 9 (Fig. S6B). After subtracting the background intensity measured outside the oocyte, we computed the mean inside-to-outside intensity ratio $I_{\text{in}}/I_{\text{out}} = 78.15$ at stage 9 (Fig. S6C).

Using this ratio, we determine the interaction parameter χ and the *oskar* volume fractions inside and outside the droplet, ϕ_{in} and ϕ_{out}, by solving Eq. (36) and Eq. (37) together with $I_{\text{in}}/I_{\text{out}} = \phi_{\text{in}}/\phi_{\text{out}}$, which assumes that intensity is proportional to volume fraction. A similar approach was used in [3] to determine the thermodynamic parameters of P-granule condensation in *Caenorhabditis elegans*.

Table S2: List of parameters for the *oskar*–Staufen particles.

Parameter	N	a (nm)	A (μm^2)	h (μm)	c_0 (μM)	ϕ_0	$I_{\rm in}/I_{\rm out}$	ν/ν_0	χ	$\phi_{\rm in}$	$\phi_{\rm out}$
Value	33544	200	7333	30	760	0.0163 ± 0.0054	78.15	1.12×10^3	0.55	0.11	0.0014

7 Asymmetric growth of the oocyte

During stages 7 to 9, the oocyte grows up to fivefold in length (pole-to-pole distance from ~ 20 μm to ~ 100 μm over ~ 12 hours), predominantly from the anterior side. This corresponds to a characteristic growth speed $u_g \approx 1.9$ nm/s. Using the effective particle radius $a_{\rm eff} \approx 100$ nm determined from the photoconversion experiments in Sec. 4 and an effective cytoplasmic viscosity 1000 times that of water, we estimate the diffusive transport speed of *oskar* RNPs across the oocyte to be $u_d = k_BT/(2\pi\mu\, a_{\rm eff}\, R_{\rm oocyte}) \approx 0.13$ nm/s. Since diffusion is much slower than growth ($u_g/u_d > 10$), we incorporate the effect of asymmetric growth in two discrete steps. We first solve the problem in a smaller geometry representing the stage-7 oocyte, and then use the resulting steady state as the initial condition for a separate simulation in a geometry five times larger. The details are described below.

The simulations are performed in an axisymmetric spherical domain V_f of radius $R_f = 5$ about the z-axis. The initial condition is constructed from the steady state of a separate simulation on a smaller spherical domain V_i of radius $R_i = 1$ with uniform binding and unbinding. The main challenge is that the numerical grids of the two domains do not coincide: they have different spatial resolutions and their centers are offset. Here we describe how the solution on V_i is mapped onto the V_f grid.

The domain V_i is placed at the posterior side of V_f, its center displaced along the anterior–posterior (z) axis by $z_{\rm shift} = R_f - R_i = 4$, so that the two share a common pole (Fig. 5 of the main text). We define radial and polar coordinates measured from the center of V_i,

$$r(\boldsymbol{x}) = \sqrt{x^2 + y^2 + (z - z_{\rm shift})^2}, \qquad \boldsymbol{x} \in V_f, \tag{38}$$

$$\theta(\boldsymbol{x}) = \arccos\left(\frac{z - z_{\rm shift}}{r}\right). \tag{39}$$

For every $\boldsymbol{x} \in V_f$, we evaluate the initial volume fraction from the steady-state V_i solution at (r, θ) directly from its spectral representation,

$$\phi_{f0}(r,\theta) = \begin{cases} \phi_i(r,\theta,t\to\infty), & r(\boldsymbol{x}) \le R_i, \\ \phi_i(R_i,\theta,t\to\infty), & r(\boldsymbol{x}) > R_i, \end{cases} \tag{40}$$

where the azimuthal angle is immaterial in the axisymmetric geometry. This mapping is exact up to spectral truncation error, even though the two simulations use non-overlapping grids with different resolutions. Outside V_i, ϕ is set to a constant value ϕ_∞. To connect the interior and exterior values smoothly, we use a hyperbolic-tangent weight of half-width $\delta = 0.25$,

$$B(r) = \tfrac{1}{2}\big[1 - \tanh\big((r - R_i)/\delta\big)\big], \tag{41}$$

so that the initial condition is

$$\phi(\boldsymbol{x}, 0) = B(r)\,\phi_{f0}(r,\theta) + \big[1 - B(r)\big]\,\phi_\infty, \qquad \boldsymbol{x} \in V_f. \tag{42}$$

The weight function departs from 0 and 1 only within a thin shell around $r = R_i$, so that both the interior solution and the exterior value ϕ_∞ are recovered away from this boundary. In the simulation

of Fig. 5, we set $\phi_\infty = 0.0107$, giving an average volume fraction $\phi_0 = |V_f|^{-1} \int_{V_f} \phi(\boldsymbol{x}, 0)\,\mathrm{d}v = 0.011$. Finally, we initialize the cortical field in local equilibrium with ϕ at the boundary ($\partial_t \phi_c = 0$),

$$\phi_c(\boldsymbol{x}, 0) = \frac{\beta}{\alpha(\boldsymbol{x})}\,\phi(\boldsymbol{x}, 0), \qquad \boldsymbol{x} \in \partial V_f. \tag{43}$$

References

[1] Paul J Flory. Thermodynamics of high polymer solutions. *Journal of Chemical Physics*, 10(1):51–61, 1942.

[2] Maurice L Huggins. Solutions of long chain compounds. *Journal of Chemical Physics*, 9(5):440–440, 1941.

[3] Anatol W. Fritsch, Andrés F. Diaz-Delgadillo, Omar Adame-Arana, Carsten Hoege, Matthäus Mittasch, Moritz Kreysing, Mark Leaver, Anthony A. Hyman, Frank Jülicher, and Christoph A. Weber. Local Thermodynamics Govern Formation and Dissolution of *Caenorhabditis* elegans P Granule Condensates. *Proceedings of the National Academy of Sciences*, 118(37):e2102772118, 2021.

[4] Anthony A. Hyman, Christoph A. Weber, and Frank Jülicher. Liquid-Liquid Phase Separation in Biology. *Annual Review of Cell and Developmental Biology*, 30(1):39–58, 2014.

[5] Frank Jülicher and Christoph A. Weber. Droplet Physics and Intracellular Phase Separation. *Annual Review of Condensed Matter Physics*, 15(1):237–261, 2024.

[6] Keaton J Burns, Geoffrey M Vasil, Jeffrey S Oishi, Daniel Lecoanet, and Benjamin P Brown. Dedalus: A flexible framework for numerical simulations with spectral methods. *Physical Review Research*, 2(2):023068, 2020.

[7] J. Abate and P. P. Valkó. Multi-precision Laplace transform inversion. *International Journal for Numerical Methods in Engineering*, 60(5):979–993, 2004.

[8] Mainak Bose, Marko Lampe, Julia Mahamid, and Anne Ephrussi. Liquid-to-solid phase transition of oskar ribonucleoprotein granules is essential for their function in Drosophila embryonic development. *Cell*, 185(8):1308–1324, 2022.
